# Efficacy of indigenous Lacticaseibacillus paracasei from dadih on growth performance, hepatoprotection, and economic efficiency in broilers challenged with avian pathogenic Escherichia coli

**DOI:** 10.14202/vetworld.2026.4042-4053

**Published:** 2026-09-14

**Authors:** Widya Paramita Lokapirnasari, Aswin Rafif Khairullah, Lilik Maslachah, Efi Rokana, Amung Logam Saputro, Zulfi Nur Amrina Rosyada, Andreas Berny Yulianto, Himatul Ilma Silfia, Muhammad Aviv Firdaus, Zein Ahmad Baihaqi, Ertika Fitri Lisnanti, Tabita Dameria Marbun, Saifur Rehman, Muhammad Shakeel

**Affiliations:** 1 Division of Animal Husbandry, Faculty of Veterinary Medicine, Universitas Airlangga, Jl. Dr. Ir. H. Soekarno, Kampus C Mulyorejo, Surabaya 60115, East Java, Indonesia.; 2 Research Center for Veterinary Science, National Research and Innovation Agency (BRIN), Jl. Raya Bogor KM.46 Cibinong, Bogor 16911, West Java, Indonesia.; 3 Division of Basic Veterinary Medicine, Faculty of Veterinary Medicine, Universitas Airlangga, Jl. Dr. Ir. H. Soekarno, Kampus C Mulyorejo, Surabaya 60115, East Java, Indonesia.; 4 Division of Animal Husbandry, Faculty of Agriculture, Universitas Islam Kadiri, Jl. Sersan Suharmaji No.38, Manisrenggo, Kediri 64128, East Java, Indonesia.; 5 Veterinary Medicine Study Program, Faculty of Health, Medicine, and Life Sciences, Universitas Airlangga, Jl. Wijaya Kusuma No.113, Mojopanggung, Giri, Banyuwangi 68425, East Java, Indonesia.; 6 Faculty of Veterinary Medicine, Universitas Wijaya Kusuma Surabaya, Jl. Dukuh Kupang XXV No.54, Dukuh Kupang, Dukuh Pakis, Surabaya 60225, East Java, Indonesia.; 7 Master Program of Veterinary Agribusiness, Faculty of Veterinary Medicine, Universitas Airlangga, Jl. Dr. Ir. H. Soekarno, Kampus C Mulyorejo, Surabaya 60115, East Java, Indonesia.; 8 Research Center for Animal Husbandry, National Research and Innovation Agency (BRIN), Jl. Raya Bogor KM.46 Cibinong, Bogor 16911, West Java, Indonesia.; 9 Animal Nutrition Laboratory, Kyungpook National University, 2559, Gyeongsang-daero, Sangju-si 37224, Gyeongsangbuk-do, South Korea.; 10 Department of Pathobiology, Faculty of Veterinary and Animal Sciences, City Campus (Quaid-e-Azam Campus), Gomal University, Multan Road, Dera Ismail Khan 29050, Khyber Pakhtunkhwa, Pakistan.; 11 Department of Clinical Studies, Faculty of Veterinary and Animal Sciences, PMAS-Arid Agriculture University Rawalpindi, Shamsabad, Murree Road, Rawalpindi 46000, Punjab, Pakistan.

**Keywords:** antibiotic growth promoter alternative, avian pathogenic Escherichia coli, dadih, economic efficiency, hepatoprotection, Lacticaseibacillus paracasei, probiotics, zero hunger

## Abstract

**Background and Aim:**

Avian pathogenic *Escherichia coli* (APEC) is a major cause of colibacillosis in broiler chickens, leading to impaired growth, liver dysfunction, and substantial economic losses. Increasing restrictions on antibiotic growth promoters (AGPs) due to antimicrobial resistance have intensified the search for sustainable alternatives. Indigenous probiotics isolated from traditional fermented foods may provide effective region-specific solutions. This study evaluated the efficacy of indigenous *Lacticaseibacillus* *paracasei* isolated from dadih on growth performance, carcass traits, hepatoprotection, and economic efficiency in broilers challenged with APEC and compared its effectiveness with that of a conventional AGP.

**Materials and Methods:**

Eighty one-day-old male broiler chickens were randomly assigned to four treatment groups with five replicates of four birds each: uninfected control (T0), APEC-infected control (T1), AGP-treated (zinc bacitracin; T2), and probiotic-treated (*L. paracasei* 153 Vi; T3). Birds were orally challenged with APEC (1.5 × 10⁸ colony-forming units [CFU]/mL) at 21 days of age. The probiotic was administered through drinking water (5 mL/L; 1.2 × 10⁹ CFU/mL), whereas zinc bacitracin was incorporated into feed (1 g/kg). Feed and nutrient intake, final body weight, carcass characteristics, serum glutamate pyruvate transaminase (SGPT), serum glutamate oxaloacetate transaminase (SGOT), and Income Over Feed Cost (IOFC) were evaluated. Data were analyzed using one-way analysis of variance followed by Duncan’s Multiple Range Test at p < 0.05.

**Results:**

The AGP group exhibited the highest feed and nutrient intake. However, the probiotic group achieved the greatest final body weight (1993.0 g), carcass weight (1337.8 g), and carcass percentage (68.75%) (p < 0.05). *L. paracasei* supplementation markedly reduced SGPT (105.07 U/L) and SGOT (59.05 U/L) compared with the infected control, demonstrating superior hepatoprotective activity. Economic analysis showed that the probiotic treatment generated the highest IOFC at both experimental and farm scales, substantially exceeding the AGP-treated and infected control groups.

**Conclusion:**

Indigenous *L. paracasei* isolated from dadih effectively improved growth performance, carcass yield, liver function, and economic returns in APEC-challenged broilers, outperforming the conventional AGP under the conditions of this study. These findings support its potential as a safe, sustainable, and economically viable alternative to AGPs for improving poultry productivity while contributing to strategies aimed at reducing antimicrobial use in commercial broiler production.

## INTRODUCTION

Avian pathogenic *Escherichia coli* (APEC) is one of the most important bacterial pathogens causing colibacillosis in poultry and represents a major challenge to the global poultry industry [1]. APEC infection is associated with increased morbidity and mortality, impaired growth performance, and substantial economic losses [2]. In broiler chickens, infection commonly induces intestinal inflammation, reduced nutrient absorption, decreased body weight gain, and pathological damage to vital organs, particularly the liver [3]. Hepatic injury is typically reflected by elevated serum glutamate pyruvate transaminase (SGPT) and serum glutamate oxaloacetate transaminase (SGOT) activities [4]. The considerable physiological and economic consequences of APEC infection highlight the need for alternative strategies that enhance disease resistance while maintaining optimal production performance [5].

Antibiotics have traditionally been used as antibiotic growth promoters (AGPs) to improve production efficiency and control bacterial diseases in poultry [6]. However, the growing prevalence of antimicrobial resistance (AMR) and increasing concerns about antibiotic residues in poultry products have led to stricter regulations governing AGP use worldwide [7]. Consequently, the poultry industry has intensified efforts to identify safe, sustainable, and effective alternatives that do not compromise animal or public health [8]. Among the available alternatives, probiotics, particularly lactic acid bacteria (LAB), have attracted considerable attention because they can improve intestinal health and production performance [9].

LAB contribute to gastrointestinal health through multiple mechanisms, including modulating the intestinal microbiota, enhancing host immune responses, producing antimicrobial metabolites, maintaining intestinal barrier integrity, and improving nutrient utilization [10, 11]. These beneficial effects enable broilers to better withstand infectious challenges, including APEC infection [12]. Among LAB, *Lacticaseibacillus* *paracasei* has demonstrated strong antagonistic activity against enteric pathogens, high tolerance to acidic and bile environments, and excellent adhesion to the intestinal epithelium, making it a promising probiotic candidate for poultry production [13].

Traditional Indonesian fermented foods, particularly dadih from West Sumatra, constitute valuable reservoirs of indigenous probiotic microorganisms, including *L. paracasei* [14]. Dadih undergoes spontaneous fermentation mediated by indigenous microbial communities that are naturally adapted to tropical environmental conditions [15]. LAB isolated from dadih exhibit high viability, desirable probiotic characteristics, and broad-spectrum antimicrobial activity against pathogenic microorganisms [16]. Consequently, these indigenous LAB represent an economical and locally available resource with considerable potential to improve broiler health and productivity under bacterial challenge [17].

Compared with imported commercial probiotic strains, indigenous probiotics isolated from traditional fermented foods may provide additional advantages through adaptation to local environmental conditions, regional microbial ecosystems, and production systems [10, 12]. Such characteristics may enhance their survival, colonization, and functional efficacy under tropical poultry production conditions. Moreover, using locally sourced probiotic strains supports sustainable livestock production by reducing dependence on imported feed additives while promoting the use of indigenous biological resources.

Beyond improving intestinal health and immunity, probiotics positively influence several economically important production parameters, including nutrient utilization, growth performance, carcass yield, and feed efficiency [18]. Their hepatoprotective properties may also reduce SGPT and SGOT activities by alleviating hepatic injury associated with APEC infection [19]. Collectively, these improvements enhance production efficiency and profitability, which can be evaluated using Income Over Feed Cost (IOFC), an important indicator of economic performance in commercial broiler production [20].

Although numerous studies have demonstrated the beneficial effects of probiotics in poultry, relatively few have evaluated indigenous *L. paracasei* isolated from dadih under APEC challenge conditions. Furthermore, previous investigations have primarily focused on intestinal health, immune responses, antioxidant activity, or hematological parameters, whereas comprehensive evaluations integrating growth performance, nutrient intake, carcass characteristics, hepatoprotective effects, and economic efficiency remain limited. Information comparing indigenous *L. paracasei* directly with conventional AGPs under controlled APEC challenge conditions is particularly scarce. Consequently, the practical production value and economic feasibility of this indigenous probiotic as an alternative to AGPs have not been fully established.

Therefore, this study aimed to evaluate the efficacy of indigenous *L. paracasei* isolated from dadih as a sustainable alternative to AGPs in broiler chickens experimentally challenged with APEC. Specifically, the study assessed its effects on nutrient intake, growth performance, carcass characteristics, liver function as determined by SGPT and SGOT activities, and economic performance using IOFC. In addition, this study extends the authors' previous work reported by Lokapirnasari *et al.* [13], which demonstrated the beneficial effects of the same *L. paracasei* isolate on antioxidant status, lipid metabolism, and hematological parameters in APEC-challenged broilers. This investigation provides new evidence on production performance, hepatoprotection, and economic efficiency, strengthening the scientific basis for using indigenous *L. paracasei* from dadih as a safe, effective, and sustainable probiotic for commercial broiler production.

## MATERIALS AND METHODS

### Ethical approval

All experimental procedures involving broiler chickens were reviewed and approved by the Animal Ethics Commission, Faculty of Veterinary Medicine, Universitas Airlangga, Surabaya, Indonesia, under Ethical Clearance No. 1.KEH.017.01.2024. The study was conducted in accordance with the institutional guidelines and accepted principles for the ethical care, housing, handling, and use of animals in research. The experimental protocol involved 80 one-day-old male broiler chickens allocated to four treatment groups, including an experimental challenge with APEC, administration of indigenous *Lacticaseibacillus* *paracasei*, and comparison with zinc bacitracin. Throughout the experimental period, appropriate husbandry and biosecurity practices were maintained, and feed and drinking water were provided *ad libitum*. Measures were taken to minimize animal stress, pain, discomfort, and unnecessary suffering during housing, APEC challenge, treatment administration, handling, blood collection, and other experimental procedures. Birds were monitored for clinical manifestations following the APEC challenge, including changes in appetite, activity, and other signs associated with infection. Blood sampling was performed from the brachial vein at 35 days of age using appropriate handling and sample-collection procedures. All animal procedures were performed only to the extent necessary to achieve the scientific objectives of the study, with due consideration given to animal welfare and the principles of responsible use of animals in biomedical and veterinary research.

### Study period and location

The study was conducted between June and October 2024 at the Faculty of Veterinary Medicine, Universitas Airlangga, Surabaya, East Java, Indonesia. The experimental challenge used APEC strain O-78 [46] at 1.5 × 10⁸ colony-forming units [CFU]/mL and indigenous *L. paracasei* 153 Vi at 1.2 × 10⁹ CFU/mL. The study utilized commercial broiler feed (CP-511; PT Charoen Pokphand Indonesia Tbk., Jakarta, Indonesia), zinc bacitracin (Rojo Koyo Group, Kediri, Indonesia), male broiler chickens, battery cages (35 × 20 × 45 cm), and digital weighing scales for feed and body weight measurements.

### Study design

A total of 80 one-day-old male broiler chicks were used in this study. The experiment followed a completely randomized design with four treatment groups, five replicates per treatment, and four birds per replicate. The experimental equipment included 1- and 15-mL syringes, modified 1.5-L drinking bottles, masks, gloves, labels, hand sprayers, feed and water containers, cage sanitation equipment, garbage bags, a Gasolec™ S8 heating system (Gasolec B.V., Abbekerk, the Netherlands), postal cages (250 × 200 × 200 cm) for brooding, battery cages (35 × 20 × 45 cm), and digital weighing scales, which were also used during the experiment.

The treatments were designed to evaluate the efficacy of indigenous *L. paracasei* administered through drinking water compared with AGP supplementation through feed under APEC challenge. The treatment groups were as follows:

1. T0 = Negative control (uninfected and untreated)

2. T1 = Positive control (APEC-infected without treatment)

3. T2 = AGP (zinc bacitracin, 1 g/kg feed, APEC-infected)

4. T3 = Probiotic (*L. paracasei*, 5 mL/L drinking water, APEC-infected)

Broiler chicks were obtained from a certified commercial hatchery (MB-202; PT Japfa Comfeed Indonesia Tbk., Sidoarjo, Indonesia). Birds were reared in postal cages from 1 to 14 days of age and had received routine vaccinations from the hatchery before arrival. At 14 days of age, the birds were transferred to battery cages and maintained under identical management conditions until the experiment was completed.

### Feeding and drinking management

Broilers were fed twice daily at 08:00 and 15:00 h. The commercial broiler diet (CP-511; PT Charoen Pokphand Indonesia Tbk., Jakarta, Indonesia) contained 85.51% dry matter, 6.90% ash, 20.69% crude protein, 6.87% crude fat, 1.85% crude fiber, 49.19% nitrogen-free extract, and 2,998.04 kcal/kg metabolizable energy. Feed and drinking water were provided *ad libitum* throughout the experimental period. Probiotic supplementation was administered through the drinking water of birds assigned to T3.

### Administration of APEC, probiotic, and AGP

At 21 days of age, broilers in the infected groups were orally challenged with 0.5 mL of the APEC suspension per bird. Successful infection was confirmed clinically by the appearance of characteristic signs, including pallor of the comb and face, sneezing, reduced appetite, weakness, and diarrhea in the infected birds.

The probiotic culture was propagated in de Man, Rogosa, and Sharpe (MRS) broth (Merck KGaA, Darmstadt, Germany; Cat. No. 1.10661.0500) at 37°C for 24 h. Bacterial cells were harvested and adjusted to a final concentration of 1.2 × 10⁹ CFU/mL using the plate-counting method. The probiotic suspension was administered through drinking water at a dose of 5 mL/L [21]. Based on average daily water consumption, probiotic intake was estimated at approximately 6 × 10⁶-8 × 10⁶ CFU/bird/day. Zinc bacitracin (Rojo Koyo Group, Kediri, Indonesia) was incorporated into the feed at 1 g/kg before feeding.

### Data collection

**Feed intake and nutrient intake:** Feed intake was recorded daily by subtracting the remaining feed from the amount offered. Nutrient intake, including dry matter, organic matter, ash, crude protein, crude fat, and crude fiber, was calculated using the corresponding nutrient composition of the commercial diet. Production parameters, including final body weight, carcass weight, feed intake, and carcass percentage, were determined at 35 days of age [22].

**SGPT and SGOT analysis:** Blood samples were collected from the brachial vein at 35 days of age using non-anticoagulant blood collection tubes. After clot formation, samples were centrifuged at 1,509 × g for 10-15 min to obtain serum. Serum SGPT and SGOT activities were determined using an automated biochemical analyzer (Reflotron® Plus; Roche Diagnostics GmbH, Mannheim, Germany) with Reflotron GPT test strips (Cat. No. 10745138) and Reflotron GOT test strips (Cat. No. 10745120), according to the method described by Tugiyanti *et al.* [23].

**IOFC:** Economic efficiency was evaluated using IOFC, calculated as the difference between revenue generated from broiler sales and the total feed cost incurred during the production period.

### Statistical analysis

Statistical analyses were performed using IBM SPSS Statistics version 27 (IBM Corp., Armonk, NY, USA). Data normality and homogeneity of variance were assessed using the Kolmogorov-Smirnov and Levene's tests, respectively. Treatment effects were analyzed using one-way analysis of variance (ANOVA) followed by Duncan's Multiple Range Test (DMRT). Differences were considered statistically significant at p < 0.05.

SGPT and SGOT activities were expressed as mean ± standard deviation (SD) and analyzed using one-way ANOVA followed by DMRT. IOFC was similarly compared among treatment groups using one-way ANOVA. Pearson correlation analysis was performed to evaluate associations among probiotic supplementation, feed intake, carcass characteristics, and liver enzyme activities. Effect sizes were expressed as partial eta-squared (η²). Graphical presentations were generated using GraphPad Prism version 9.0 (GraphPad Software, San Diego, CA, USA).

## RESULTS

### Feed intake and nutrient intake

The effects of *L. paracasei* supplementation on feed and nutrient intake are presented in Table 1.

**Table 1:** Feed and nutrient intake of broilers in different treatment groups.

**Parameters**	**T0**	**T1**	**T2**	**T3**
Feed intake (g/head/day)	82.45ᵃ ± 3.40	82.31ᵃ ± 2.75	88.50ᵇ ± 3.01	83.70ᵃ ± 2.55
DM intake (g/head/day)	70.51ᵃ ± 2.91	70.39ᵃ ± 2.35	75.67ᵇ ± 2.57	71.57ᵃ ± 2.18
Ash intake (g/head/day)	5.69ᵃ ± 0.23	5.68ᵃ ± 0.18	6.11ᵇ ± 0.20	5.77ᵃ ± 0.17
Organic matter intake (g/head/day)	76.77ᵃ ± 3.16	76.63ᵃ ± 2.56	82.39ᵇ ± 2.80	77.92ᵃ ± 2.37
Crude protein intake (g/head/day)	17.06ᵃ ± 0.71	17.03ᵃ ± 0.57	18.31ᵇ ± 0.62	17.31ᵃ ± 0.53
Crude fat intake (g/head/day)	5.67ᵃ ± 0.23	5.65ᵃ ± 0.19	6.08ᵇ ± 0.21	5.75ᵃ ± 0.17
Crude fiber intake (g/head/day)	1.53ᵃ ± 0.06	1.53ᵃ ± 0.05	1.64ᵇ ± 0.06	1.55ᵃ ± 0.04
Nitrogen-free extract intake (g/head/day)	40.56ᵃ ± 1.67	40.50ᵃ ± 1.35	43.53ᵇ ± 1.48	41.17ᵃ ± 1.25
Metabolizable energy intake (kcal/head/day)	247.21ᵃ ± 10.20	246.78ᵃ ± 8.25	298.31ᵇ ± 11.25	250.94ᵃ ± 7.65
Carbohydrate intake (g/head/day)	42.09ᵃ ± 1.74	42.02ᵃ ± 1.40	45.18ᵇ ± 1.54	42.73ᵃ ± 1.30

Values are presented as mean ± SD. Different superscript letters (ᵃ, ᵇ) within the same row indicate significant differences among treatment groups (p < 0.05). DM = Dry matter; ME = Metabolizable energy; SD = Standard deviation.

Broilers in the T2 (AGP) group exhibited the highest feed intake (88.50 g/head/day) and dry matter intake (75.67 g/head/day), both of which were significantly higher than those in the T0, T1, and T3 groups (p < 0.05). Similar trends were observed for ash (6.11 g/head/day), organic matter (82.39 g/head/day), crude protein (18.31 g/head/day), crude fat (6.08 g/head/day), and crude fiber (1.64 g/head/day), with T2 consistently recording the greatest nutrient intake (p < 0.05).

Nitrogen-free extract intake was also highest in T2, suggesting greater energy substrate consumption. Although ME intake differed significantly among treatments (p < 0.05), the variation among groups was relatively small. Carbohydrate intake followed a comparable pattern, with broilers receiving AGP supplementation consuming the greatest amount.

Overall, AGP supplementation promoted the highest feed and nutrient intake, whereas *L. paracasei* supplementation (T3) maintained nutrient intake at levels comparable to or greater than those of the control groups while achieving superior production performance, indicating more efficient nutrient utilization.

### Growth performance and carcass traits

The effects of *L. paracasei* supplementation on growth performance and carcass characteristics are presented in Table 2.

**Table 2:** Final body weight, carcass weight, and carcass percentage of broilers in different treatment groups.

**Parameters**	**T0**	**T1**	**T2**	**T3**
Final body weight (g)	1798.20ᵇ ± 59.15	1671.00ᵃ ± 28.90	1723.00ᵃᵇ ± 57.15	1993.00ᶜ ± 20.86
Carcass weight (g)	1108.24ᵃ ± 58.69	1093.36ᵃ ± 25.18	1120.88ᵃ ± 31.35	1337.80ᵇ ± 36.80
Carcass percentage (%)	63.74ᵃᵇ ± 1.51	63.45ᵃ ± 2.43	65.59ᵇ ± 0.47	68.75ᶜ ± 0.17

Values are presented as mean ± SD. Different superscript letters (ᵃ-ᶜ) within the same row indicate significant differences among treatment groups (p < 0.05). SD = Standard deviation.

Broilers supplemented with *L. paracasei* (T3) achieved the greatest final body weight (1,993.00 g), significantly higher than all other treatment groups (p < 0.05). Birds in the T2 (AGP) group exhibited an intermediate final body weight (1,723.00 g), which was significantly greater than that of the infected untreated group (T1) but remained significantly lower than that of T3.

A similar trend was observed for carcass characteristics. The T3 group produced the highest carcass weight (1,337.80 g) and carcass percentage (68.75%), both of which were significantly greater than those recorded in the T0, T1, and T2 groups (p < 0.05). These findings demonstrate that *L. paracasei* supplementation improved both growth performance and carcass yield more effectively than AGP supplementation.

Direct comparison between the probiotic and AGP treatments further demonstrated the superiority of *L. paracasei*. Final body weight was significantly greater in T3 than in T2 (p = 0.003), while carcass percentage was also significantly higher in T3 (p = 0.012), indicating improved production performance in probiotic-supplemented broilers.

### Liver function

The effects of the treatments on liver function are presented in Table 3. Significant differences in SGPT and SGOT activities were observed among the treatment groups (p < 0.05). The T0 (negative control) group exhibited the lowest SGPT activity (66.91 U/L), consistent with normal liver function. In contrast, broilers in the T1 group (APEC-infected without treatment) showed the highest SGPT activity (159.12 U/L), indicating marked hepatocellular injury. Supplementation with either AGP (T2) or *L. paracasei* (T3) markedly reduced SGPT activity to 122.56 U/L and 105.07 U/L, respectively, demonstrating attenuation of hepatic damage. The reduction was more pronounced in the probiotic-treated birds than in the AGP-treated birds.

**Table 3:** SGPT and SGOT activities of broilers in different treatment groups.

**Variables**	**T0**	**T1**	**T2**	**T3**
SGPT (U/L)	66.91ᵃ ± 5.89	159.12ᵈ ± 9.08	122.56ᶜ ± 4.46	105.07ᵇ ± 10.16
SGOT (U/L)	78.31ᵇ ± 1.39	143.26ᵈ ± 6.74	128.91ᶜ ± 4.83	59.05ᵃ ± 7.18

Values are presented as mean ± SD. Different superscript letters (ᵃ-ᵈ) within the same row indicate significant differences among treatment groups (p < 0.05). SGPT = Serum glutamate pyruvate transaminase; SGOT = Serum glutamate oxaloacetate transaminase; SD = Standard deviation.

A comparable pattern was observed for SGOT activity. Broilers supplemented with *L. paracasei* exhibited the lowest SGOT activity (59.05 U/L), whereas the untreated infected group recorded the highest value (143.26 U/L), confirming severe hepatic impairment following APEC infection. The T0 and T2 groups exhibited intermediate SGOT activities of 78.31 U/L and 128.91 U/L, respectively.

Interestingly, SGOT activity in T3 was lower than that observed in the uninfected control group (T0). This finding suggests that *L. paracasei* may exert a pronounced hepatoprotective effect beyond merely preventing APEC-induced liver injury, although the contribution of normal biological variation among individual birds cannot be excluded.

Collectively, these findings indicate that *L. paracasei* supplementation effectively alleviated hepatic stress associated with APEC infection and provided greater hepatoprotection than AGP supplementation.

### Economic performance

The effects of the treatments on economic performance, expressed as IOFC, are presented in Table 4 and Figure 1.

**Table 4:** IOFC of broilers in different treatment groups.

**Variables**	**T0**	**T1**	**T2**	**T3**
**Study scale (10 ** **birds** **/treatment group)**				
IOFC (IDR)	11,143.80ᵇ ± 3,776.94	6,960.00ᵃᵇ ± 4,323.05	3,245.60ᵃ ± 2,858.36	16,812.20ᶜ ± 3,840.45
**Commercial scale (1,000 ** **birds** **/treatment group)**				
IOFC (IDR)	1,114,418.00ᵇ ± 377,693.79	696,038.00ᵃᵇ ± 432,305.13	324,545.40ᵃ ± 285,854.70	1,681,235.00ᶜ ± 384,034.64

Values are presented as mean ± SD. Different superscript letters (ᵃ-ᶜ) within the same row indicate significant differences among treatment groups (p < 0.05). IOFC = Income over feed cost; IDR = Indonesian rupiah; SD = Standard deviation.

At the experimental scale (10 birds per treatment), the T2 (AGP) group produced the lowest IOFC (Indonesian rupiah [IDR] 3,245.60), indicating the poorest economic efficiency. The untreated infected group (T1) generated a moderate IOFC (IDR 6,960.00), whereas the uninfected control group (T0) achieved a substantially higher IOFC (IDR 11,143.80). In contrast, broilers supplemented with *L. paracasei* (T3) produced the highest IOFC, which was significantly greater than those of both T2 (p = 0.001) and T1 (p = 0.004), demonstrating the clear economic benefit of probiotic supplementation (Figure 1).

**Figure 1:**
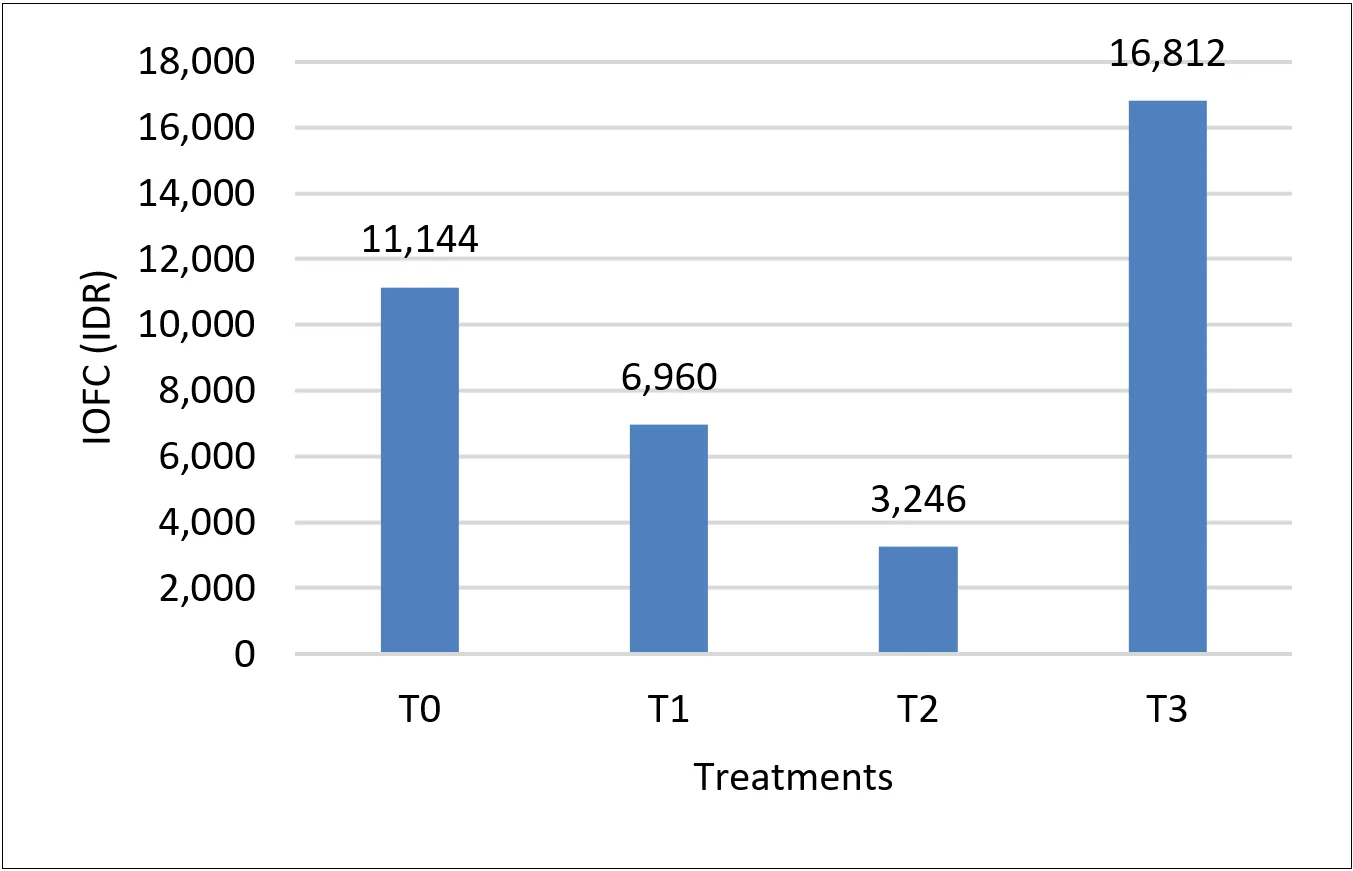
Income Over Feed Cost (IOFC) of broilers in different treatment groups. Broilers supplemented with *L. paracasei* (T3) exhibited the highest IOFC, whereas Antibiotic growth promoter supplementation (T2) resulted in the lowest economic return.

When extrapolated to a commercial production scale of 1,000 birds, the same trend was maintained. The T2 group again generated the lowest IOFC (IDR 324,545.40), followed by T1 (IDR 696,038.00). The T0 group yielded an IOFC of IDR 1,114,418.00, while T3 achieved the highest profitability, with an IOFC of IDR 1,681,235.00.

The absence of mortality throughout the experimental period indicates that differences in IOFC were primarily attributable to improvements in production performance and feed utilization rather than survival rate. Overall, *L. paracasei* supplementation provided the greatest economic return, outperforming both AGP supplementation and the untreated groups, thereby supporting its potential as a sustainable alternative to AGPs in commercial broiler production.

## DISCUSSION

### Feed intake and nutrient intake

The present study evaluated the effects of indigenous *L. paracasei* isolated from dadih on nutrient intake, growth performance, carcass characteristics, liver function, and economic efficiency in broilers challenged with APEC. The findings clearly demonstrate that *L. paracasei* supplementation provides substantial biological and economic benefits, producing superior outcomes to AGP supplementation under the experimental conditions. These findings support the potential of this indigenous probiotic as a safe, sustainable, and effective alternative to AGPs in commercial broiler production.

Broilers receiving AGP supplementation (T2) exhibited the highest feed and nutrient intake across most measured parameters, including dry matter, crude protein, crude fat, and carbohydrate intake. These findings are consistent with the established role of AGPs in modulating intestinal microbiota, suppressing subclinical bacterial infections, and improving digestive efficiency, thereby stimulating feed consumption [24]. In contrast, broilers supplemented with *L. paracasei* (T3) maintained a more balanced feed intake while achieving superior production performance, indicating greater nutrient utilization efficiency rather than increased feed consumption [12].

The ability of *L. paracasei* to stabilize intestinal microbial communities likely contributed to maintaining nutrient digestibility during APEC challenge [25]. LAB produce organic acids, bacteriocins, and digestive enzymes that facilitate feed degradation, inhibit pathogenic colonization, and preserve intestinal integrity [26]. Consequently, the greater feed intake observed in the AGP group, despite its lower growth performance and economic return, may reflect reduced feed utilization efficiency or increased maintenance requirements under pathogen challenge. Although nutrient intake in T3 did not exceed that of T2, the improved production performance achieved with moderate feed consumption indicates enhanced metabolic efficiency [27]. Such improved feed utilization is particularly advantageous in commercial poultry production, where feed represents the largest component of production costs.

### Growth performance and carcass traits

Broilers supplemented with *L. paracasei* (T3) exhibited the greatest improvements in final body weight, carcass weight, and carcass percentage. These findings demonstrate the beneficial effects of *L. paracasei* on intestinal function, nutrient utilization, and overall metabolism [28]. Previous studies have shown that LAB improve intestinal morphology by increasing villus height, modulating immune responses, and reducing intestinal inflammation, thereby enhancing nutrient absorption and promoting growth [29].

Conversely, broilers in the untreated APEC-infected group (T1) exhibited marked growth suppression, consistent with intestinal inflammation, impaired nutrient absorption, and reduced metabolic efficiency associated with APEC infection [30]. Although AGP supplementation partially restored growth performance, the improvements remained inferior to those achieved with *L. paracasei* [31]. These observations support accumulating evidence that probiotics can match or even surpass AGPs in promoting broiler growth, particularly under infectious conditions [32].

The superior carcass yield observed in T3 further suggests enhanced protein deposition and more efficient nutrient partitioning [33]. Improved intestinal health reduces the metabolic cost of immune activation and inflammatory responses, allowing a greater proportion of nutrients to be directed toward muscle accretion [34]. Because carcass yield directly influences meat production efficiency and commercial profitability, these improvements provide substantial practical value for poultry producers [35].

### Liver function

APEC infection induces systemic inflammation and hepatocellular injury, resulting in elevated SGPT and SGOT activities [5]. This pathological response was clearly demonstrated in the untreated infected group (T1), which exhibited the highest enzyme activities. In contrast, broilers supplemented with *L. paracasei* (T3) showed markedly reduced SGPT activity and the lowest SGOT activity, indicating substantial hepatoprotection during APEC infection [36].

Several mechanisms may contribute to this protective effect. Probiotics suppress pathogen proliferation, enhance antioxidant defenses, and regulate systemic immune responses [37]. LAB produce bioactive metabolites, including short-chain fatty acids (SCFAs) and antioxidant compounds, which reduce oxidative stress and protect hepatic tissues from inflammatory damage [38]. Furthermore, *L. paracasei* may improve intestinal barrier integrity, reduce endotoxin translocation, and increase SCFA production, thereby attenuating hepatic inflammation and preserving liver function. Probiotics have also been reported to suppress pro-inflammatory cytokine production, thereby reducing hepatic metabolic burden and limiting hepatocellular injury [39]. Although AGP supplementation also reduced liver enzyme activities, its hepatoprotective effects remained less pronounced than those observed with *L. paracasei* [40].

Collectively, these findings indicate that *L. paracasei* exerts broader physiological benefits than conventional AGPs by combining antimicrobial activity with immunomodulatory, antioxidant, and metabolic regulatory functions [41].

### Economic performance

Economic evaluation demonstrated that *L. paracasei* supplementation produced the highest IOFC at both the experimental and commercial production scales, whereas AGP supplementation generated the lowest economic return. The superior profitability associated with probiotic supplementation resulted from the combined effects of efficient feed utilization, improved growth performance, enhanced carcass yield, and reduced physiological stress [42].

In contrast, despite exhibiting the highest feed intake, broilers receiving AGP supplementation achieved only moderate body weight gain, resulting in increased feed costs relative to production output [43]. Consequently, AGP supplementation yielded lower IOFC values, illustrating the declining economic benefit of AGPs under modern poultry production systems characterized by increasing antimicrobial restrictions and growing consumer demand for antibiotic-free poultry products [44].

The consistently greater economic performance observed in the probiotic group highlights the commercial value of indigenous *L. paracasei* as a sustainable feed additive. These findings are particularly relevant to tropical poultry production systems, where locally adapted LAB strains may exhibit enhanced survival, colonization, and functional efficacy under regional environmental conditions [45].

### Implications for sustainable poultry production

The present findings demonstrate that indigenous *L. paracasei* isolated from dadih provides comparable or superior benefits to AGPs in growth performance, liver health, carcass yield, and economic efficiency. Using indigenous probiotic strains also supports the development of region-specific nutritional strategies better adapted to local environmental conditions and microbial ecosystems [46, 47].

Importantly, the present study extends our previous work reported by Lokapirnasari *et al.* [48]. While the earlier study demonstrated improvements in antioxidant status, lipid metabolism, and hematological parameters using the same *L. paracasei* isolate, the present investigation provides new evidence regarding nutrient utilization, production performance, hepatoprotection, carcass characteristics, and economic efficiency. Together, these complementary findings provide a more comprehensive understanding of the biological and practical value of this indigenous probiotic in broiler production.

Given the increasing global restrictions on AGP use, LAB-based probiotics represent an effective strategy for maintaining poultry health, improving production efficiency, and enhancing food safety [49]. Furthermore, utilizing indigenous probiotic strains supports sustainable livestock production by reducing dependence on imported feed additives while promoting the conservation and application of local microbial biodiversity [50].

## CONCLUSION

The present study demonstrated that supplementation with indigenous *L. paracasei* isolated from dadih effectively improved broiler performance during APEC challenge. Although AGP supplementation resulted in the highest feed and nutrient intake, *L. paracasei* achieved superior nutrient utilization efficiency, as reflected by the greatest final body weight, carcass weight, carcass percentage, and IOFC. Furthermore, probiotic supplementation markedly reduced SGPT and SGOT activities compared with the untreated infected group and provided greater hepatoprotection than AGP supplementation, indicating its ability to alleviate hepatic damage associated with APEC infection.

From a practical perspective, these findings demonstrate that indigenous *L. paracasei* can serve as an effective and economically viable alternative to AGPs in broiler production. Improved growth performance, enhanced carcass yield, better liver health, and increased profitability collectively support its application in commercial poultry production, particularly in tropical regions where locally adapted probiotic strains may exhibit superior functionality. The use of indigenous probiotics also aligns with current global efforts to reduce antibiotic use and promote sustainable, antibiotic-free poultry production.

A major strength of this study is the comprehensive evaluation of biological and economic responses under an experimentally induced APEC challenge. By integrating nutrient intake, growth performance, carcass characteristics, liver function, and economic efficiency, the study provides a broader assessment of the production value of indigenous *L. paracasei*. Moreover, the findings complement the authors' previous work by extending the evidence beyond antioxidant status and hematological responses to include production-related and hepatoprotective outcomes.

Nevertheless, this study has several limitations. The experiment evaluated only a single probiotic strain and dosage under controlled experimental conditions and did not investigate changes in intestinal microbiota composition, intestinal morphology, immune responses, oxidative stress biomarkers, or molecular mechanisms underlying the observed hepatoprotective effects. In addition, the relatively short production period limits assessment of the long-term persistence of probiotic benefits.

Future studies should investigate different supplementation levels and administration strategies of indigenous *L. paracasei* under commercial farming conditions involving larger bird populations and diverse management systems. Further research integrating gut microbiome analysis, intestinal histomorphology, immune and inflammatory biomarkers, antioxidant status, and molecular pathways would provide a more comprehensive understanding of the mechanisms responsible for the probiotic's beneficial effects. Comparative studies involving multiple indigenous probiotic strains and combinations with other non-antibiotic feed additives may also facilitate the development of optimized antibiotic-free feeding strategies.

In conclusion, indigenous *L. paracasei* isolated from dadih represents a promising, safe, and sustainable probiotic that improves production performance, supports liver health, and enhances economic returns in APEC-challenged broilers. These findings provide strong scientific evidence supporting the use of locally derived probiotics as practical alternatives to AGPs for sustainable commercial poultry production.

## DATA AVAILABILITY

All data generated and analyzed during this study are included in the published article. Additional data supporting the findings of this study are available from the corresponding author upon reasonable request.

## GENERATIVE AI DECLARATION

The authors declare that generative artificial intelligence (AI) tools were used solely to improve language, grammar, and readability during manuscript preparation. All scientific content, data analysis, interpretation of results, and conclusions were developed and verified by the authors. The authors take full responsibility for the accuracy, integrity, and originality of the work presented, and no AI tool was listed as an author.

## AUTHORS’ CONTRIBUTIONS

WPL, LM, and ABY: Conceptualization, study design, supervision, project administration, and writing—original draft preparation. ALS, ER, and MAF: Data curation, investigation, formal analysis, and methodology. ZNAR and ARK: Investigation, data curation, visualization, and validation. EFL and ZAB: Methodology, investigation, and validation. HIS and TDM: Validation, formal analysis, and data interpretation. SR and MS: Methodology, investigation, data interpretation, supervision, writing—review and editing, critical revision of the manuscript, and final approval of the version to be published. All authors contributed to the study, participated in drafting or critically revising the manuscript, approved the final version, and agreed to be accountable for all aspects of the work.
